# Stress dose explains drought recovery in Norway spruce

**DOI:** 10.3389/fpls.2025.1542301

**Published:** 2025-03-06

**Authors:** Timo Knüver, Andreas Bär, Elias Hamann, Marcus Zuber, Stefan Mayr, Barbara Beikircher, Nadine K. Ruehr

**Affiliations:** ^1^ Institute of Meteorology and Climate Research-Atmospheric Environmental Research, Karlsruhe Institute of Technology, Garmisch-Partenkirchen, Germany; ^2^ Department of Botany, University of Innsbruck, Innsbruck, Austria; ^3^ Institute for Photon Science and Synchrotron Radiation, Karlsruhe Institute of Technology, Eggenstein-Leopoldshafen, Germany

**Keywords:** stress recovery, drought stress, tree water deficit, stress dose, tree water fluxes

## Abstract

**Introduction:**

Understanding the stress recovery of trees, particularly with respect to increasing droughts due to climate change, is crucial. An often-overlooked aspect is how short *versus* long drought events of high intensity (i.e., low and high stress dose) result in stress damage and affect post-stress recovery.

**Methods:**

This study examines the stress and recovery dynamics of 3-year-old *Picea abies* following a short drought (n = 5) of 18 days or a long drought (n = 9) of 51 days during late summer. We particularly assessed how the recovery of canopy conductance and tree transpiration is linked to i) stress intensity in terms of minimum water potential, ii) stress duration inferred by days below a water potential related to 12% hydraulic conductance loss (dP_12_), iii) stress dose inferred by the cumulative tree water deficit on days below P_12_ (TWD_P12_) as well as the cumulative water potential (Ψ_cum_), and iv) the percent loss of conductive xylem area (PLA).

**Results:**

Both drought treatments resulted in stem and root embolism with a higher PLA of 49% ± 10% in the long drought treatment compared to 18% ± 6% in the short drought treatment consistent across the measured plant parts. Suffering from embolism and leaf shedding (long drought, 32%; short drought, 12%), canopy conductance in the long drought treatment recovered to 41% ± 3% of the control and in the short drought treatment to 66% ± 4% at 12 days after drought release. These recovery rates were well explained by the observed PLA (R^2^ = 0.66) and the dP_12_ (R^2^ = 0.62) but best explained by stress dose metrics, particularly the cumulative TWD_P12_ (R^2^ = 0.88).

**Discussion:**

Our study highlights that stress duration and intensity should be integrated to assess post-stress recovery rates. Here, the tree water deficit derived from point dendrometers appears promising, as it provides a non-destructive and high temporal resolution of the stress dose.

## 1 Introduction

Climate change is driving more frequent and severe drought periods, with significant consequences for tree performance and survival. Globally, tree dieback has been repeatedly linked to drought in the Anthropocene (Allen et al., 2010; Anderegg et al., 2013; Nardini et al., 2013; Adams et al., 2017; Cailleret et al., 2017; Choat et al., 2018). In Central Europe, an intensification of summer droughts in recent years has profoundly impacted temperate forests, causing early leaf senescence in broadleaves and increased mortality particularly in conifers (Hari et al., 2020; Schuldt et al., 2020; Salomón et al., 2022). Often, trees did not die directly during the drought event, but drought events induced functional impairments and structural damages, resulting in tree death months to years later (Cailleret et al., 2017; Trugman et al., 2018). Ultimately, the survival and post-stress recovery of trees depend on many factors including the extent of the drought period and its intensity as well as the timing (Kannenberg et al., 2020; Ruehr and Nadal-Sala, 2025). Particularly, drought stress dose (intensity over time) may play a critical role as stress impairment and damage accumulate. Prolonged periods of water deficit consequently increase the likelihood of embolism formation and cellular damages, altering plant water transport capacities with consequences for leaf-level gas exchange rates post-drought (Rehschuh et al., 2020).

In trees, water is transported passively under tension from the soil via roots, stems, and branches to the leaves, where it is transpired via the stomata (Tyree and Zimmermann, 2002). This water transport occurs within the xylem, relying on intact, continuous, water-filled columns. Due to resistances along the transport pathway, water is transported in a metastable state under tension. During drought periods, trees initially maintain hydraulic functioning by closing stomata, which reduces water loss and delays critical tension within the xylem. With further declining soil water content and/or increasing vapor pressure deficit (VPD), trees continue to dehydrate due to cuticular water loss (Beikircher and Mayr, 2013; Blackman et al., 2023), which increases hydraulic tension and decreases plant internal water storage. This decrease in plant water storage can be measured by stem diameter shrinkage using high-resolution dendrometers and has been referred to as tree water deficit (TWD; Zweifel, 2016). Unlike water potential (Ψ) measurements, which are often invasive and non-continuous, TWD allows for continuous, non-invasive tracking of plant water status and leaf Ψ (Ziegler et al., 2024). Cavitation of xylem water columns is triggered alongside increasing xylem tensions, resulting in air-filled conduits (i.e., embolism; Tyree and Sperry, 1988). Once the first conduits are embolized, the water transport capacity declines, causing further increases in xylem tension, which may trigger further embolism spread (“runaway embolism”; Tyree and Sperry, 1988). The onset of embolism spread is commonly compared among species and organs as P_12_, the xylem pressure at which 12% of hydraulic conductivity is lost. Concurrently, P_50_ is often referred to as the xylem pressure at which structural damages occur (Brodribb and Cochard, 2009; Preisler et al., 2021), and embolism refilling post-drought has been shown to be unlikely in conifers (Hammond et al., 2019; Rehschuh et al., 2020; Mantova et al., 2022; Wagner et al., 2023). The degree to which tissues are damaged during drought depends on the intensity and duration of the drought event and may differ among plant organs (Johnson et al., 2016), with consequences for post-stress recovery. The hydraulic vulnerability segmentation hypothesis (Tyree and Ewers, 1991; Tyree and Zimmermann, 2002) suggests that there are distinct patterns of vulnerability thresholds to drought-induced xylem embolism within plants. According to this hypothesis, distal plant parts like leaves or roots are more susceptible to embolism formation compared to central and older parts such as the trunk. This segmentation may protect the central water transport structures with high carbon investments. The duration of a drought event may hence affect the degree of embolism in different tree tissues. It is likely that the longer the drought lasts, the more uniform the tissue water potential becomes alongside the embolism spread. However, the drought duration may also trigger leaf damage and result in leaf shedding, which typically increases with drought duration, mitigating water loss and embolism spread (Nadal-Sala et al., 2021; Sterck et al., 2024). Thus, embolism spread and tissue damage can critically affect the post-stress recovery capacity of trees.

The post-stress recovery rate of gas exchange and embolism repair is directly dependent on the physiological impact of the drought (Choat et al., 2018; Ruehr et al., 2019). Recent findings indicate that recovery is delayed more significantly by a prolonged drought than by a short, intense drought (Jiao et al., 2021). This suggests that recovery is impacted not solely by the minimum water potential (Ψ_min_) reached as an indication of drought intensity but by ancillary environmental factors, species-specific traits, and genetic factors, as well as physiological responses. For example, stress accumulation over time, i.e., the time spent under a critical threshold, may have a significant impact on recovery (Choat et al., 2018). The accumulation of drought stress results in continuing tissue dehydration, causing damage to living cells and resulting in loss of tissue functionality (Mantova et al., 2022). These damages, particularly to the water-conducting system of the trees, may accumulate over time with consequences for post-stress carbon uptake. An efficient recovery of gas exchange could be vital to resume normal metabolic functions and to upregulate growth in order to form new functional tissues (Ruehr et al., 2019). Hence, the combination of duration and severity, i.e., stress dose, of the drought may be decisive, as functional damages accumulate over time and consequently impair recovery processes. While some studies have successfully used drought stress dose as a cumulative stress impact metric, either in soil water potential (Zang et al., 2014b) or in growth after successive droughts (Navarro-Cerrillo et al., 2018; Gazol et al., 2020), most studies have focused on peak stress indices, like minimum Ψ. Although not widely adopted in experimental studies, integrating stress dose as a standard metric has great potential to improve our understanding of how drought length and intensity jointly shape recovery potential.

The ability of conifers to recover post-drought appears to be lower than that in angiosperms. This may be related to a lower amount of parenchyma cells, resulting in smaller carbohydrate storage pools, which are critical for tissue repair and regrowth of new functional xylem (Martínez-Vilalta et al., 2016; DeSoto et al., 2020). Additionally, conifers have a lower ability to refill water reservoirs after prolonged drought periods (Knüver et al., 2022; Salomón et al., 2022). Particularly, drought-induced leaf shedding can alter the whole tree water balance, resulting in pronounced stress legacy (Ruehr and Nadal-Sala, 2025). These physiological factors may contribute to the recent decline in Norway spruce (*Picea abies* H. Karst.) in Central Europe following bark beetle outbreaks after major drought events. Norway spruce is considered an isohydric species (Grams et al., 2021), closing stomata early during drought to reduce water loss and hence minimize hydraulic tension and runaway embolism. Nonetheless, Arend et al. (2021) reported a collapse of the hydraulic system in mature Norway spruce upon drought with rapid embolism spread after the initial onset of embolism formation. This highlights the susceptibility of Norway spruce to severe drought and raises the question of how trees can recover following drought release. While some studies have investigated the recovery ability of Norway spruce to drought (Tomasella et al., 2017; Hájíčková et al., 2021; Knüver et al., 2022; Hesse et al., 2023), a detailed assessment of the instantaneous post-drought recovery in relation to drought dose and hydraulic impairment is missing thus far.

In this study, we investigated canopy transpiration (*E*_c_) and canopy conductance (*g*_c_) of 3-year-old Norway spruce trees in response to drought and post-drought recovery. To assess potential drought impacts on recovery success, we related *E*_c_ and *g*_c_ to needle water potential and tree water deficit from high-resolution dendrometer measurements as well as embolism formation based on micro-CT scans. We subjected the trees to either a control treatment or a short or long drought treatment, which resulted in a low or high stress dose. The drought treatments differed in drought progression: in the long drought treatment, the drought was initially moderate, followed by an intense drought episode, while the trees in the short drought treatment only experienced a short intense drought episode. This resulted in similar minimum water potentials reached but differences in the accumulated stress dose, which allowed us to investigate the following hypotheses: 1) drought duration under the P_12_ threshold is a key factor in determining embolism formation. 2) Persisting embolism post-drought are non-uniformly distributed among above- and belowground organs following a short drought, but more uniformly distributed following a long drought. 3) The recovery potential of *g*_c_ and *E* cannot solely be explained by the minimum Ψ but is best accounted for by drought stress dose.

## 2 Materials and methods

### 2.1 Plant material

Juvenile (3-year-old) Norway spruce (*P. abies* H. Karst.; Baumschule Gracklauer, Gunzenhausen, Germany) trees (n = 100) were potted in November 2020, kept outside the greenhouse during winter and early spring, and transferred to the greenhouse in May 2021. Trees were planted in 5 L pots using a carbon-free potting substrate with a mixture of coarse (3–6 mm, 20 L) and fine (2–3 mm, 10 L) vermiculite and coarse (0.6–1.2 mm, 10 L) and fine (0.1–0.5 mm, 10 L) quartz sand (ratio vermiculite mixture to sand mixture 1:1), supplemented with 10 g slow-release fertilizer (Osmocote^®^ Exact 3-4M 16-9-12 + 2MgO + TE; ICL Specialty Fertilizers, Heerlen, The Netherlands). Air temperature and humidity were assessed using a CS215 sensor (Campbell Scientific Inc., Logan, UT, USA), and photosynthetically active radiation (PAR) was measured using a PQS 1 sensor (Kipp & Zonen, Delft, The Netherlands). These environmental conditions were logged half-hourly using a CR1000 data logger (Campbell Scientific, Logan, UT, USA). Relative humidity was set to 65%, and day and night temperatures were respectively set to 21°C and 14°C during acclimation and gradually increased to 25°C and 18°C from day 180 to day 194, resulting in a mean temperature of 23.9°C. During recovery [starting on day of year (DOY) 233], day and night temperatures were gradually decreased to 22°C and 15°C, respectively. Trees were watered regularly (3× 150 mL day^−1^) using an automated drip irrigation system (Rain Bird, Azusa, CA, USA) and light from the outside supplemented by growth lamps (T-agro, 400 W, Phillips, Hamburg, Germany), averaging to a PAR of 290 µmol m^−2^ s^−1^. Soil water content (SWC) was measured throughout the experiment in all pots (10HS, Meter Group, Pullman, WA, USA).

### 2.2 Experimental design

**Figure 1:**
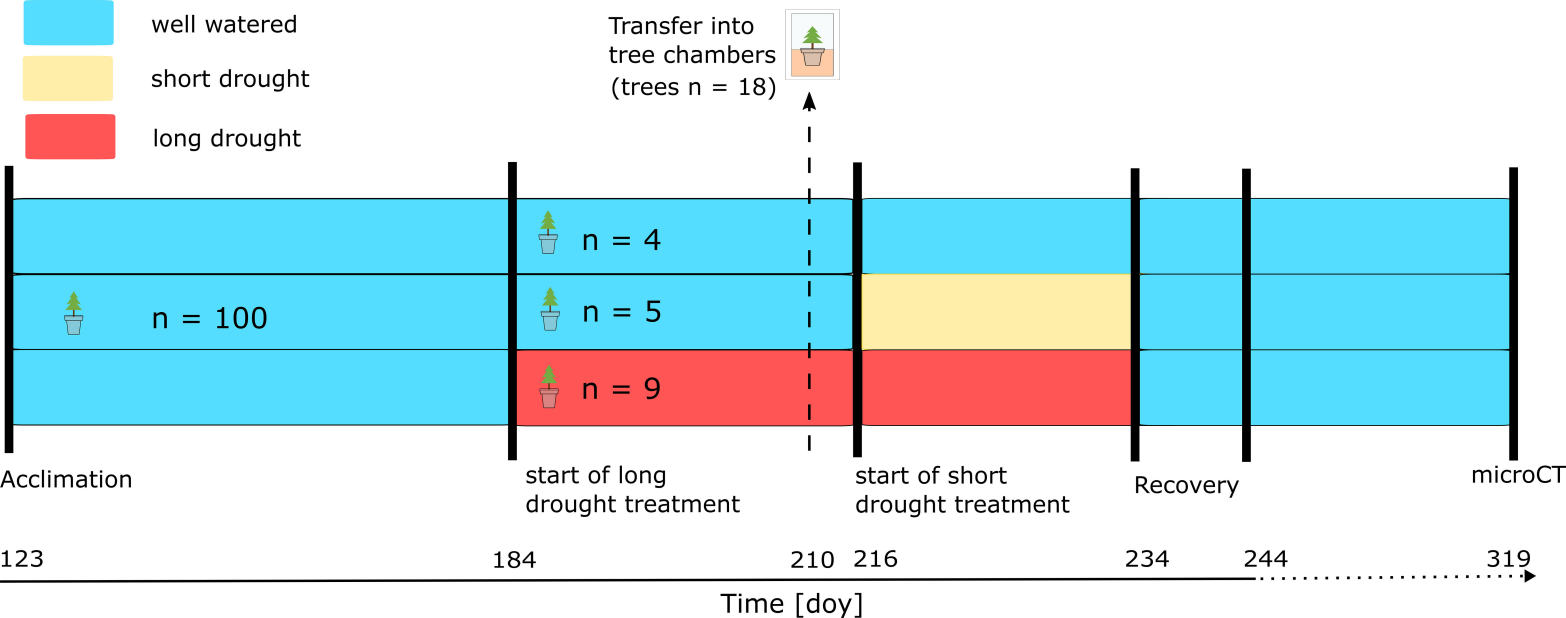
Experimental timeline. Norway spruce trees (n = 100) were transferred to the greenhouse on DOY 123 for acclimation before treatments were initiated. Trees per treatment (control, n = 4; short drought, n = 5; long drought, n = 9) were randomly selected and transferred to the tree chambers on DOY 209 to measure canopy conductance (*g*_c_) and transpiration (E) continuously on DOY 210 and onward. In the long drought (red) treatment, the trees were subjected to drought on DOY 184 first by reducing irrigation, and then on DOY 194, water was completely withheld. We watered trees of the long drought treatment on DOY 209/210 when transferring trees into the tree chambers and on DOY 214 to prevent a too rapid decline in water potential. For trees in the short drought treatment (yellow), drought was induced on DOY 216 and onward.

A subset (n = 40; long drought treatment; **Figure 1**, red) of trees was drought-treated already before the trees were transferred to the continuous gas exchange chamber system, as follows. Irrigation was reduced by 70% (3 × 45 mL/day) starting on DOY 184, and on DOYs 194–208, water was completely withheld (**Figure 1**) until a water potential was reached, which indicated the onset of embolism formation (>P_12_, see “Hydraulic vulnerability” section). Trees of the control and short drought treatment were watered during this time. As shoot development was already complete before any drought was initiated, we did not observe any new shoot development during this period. On DOY 209, trees of similar stem diameter and height were selected from each treatment (n = 4 for control, n = 5 for short drought, and n = 9 for long), which were placed into individual tree chambers as described below. On DOY 209 and DOY 210, trees of the long drought treatment were supplied with 2 × 150 mL of water to prevent a too fast decline in water potentials while transferring trees into the individual tree chambers, which was necessary to achieve similar stress intensities (lowest water potential) at the end of the drought. Trees were acclimated in the chambers for 1 day before the measurements were started on DOY 210 and onward. On DOY 216, water was completely withheld in the previously well-watered short drought treatment (**Figure 1**, yellow). On DOY 214, a few trees of the long drought treatment that showed a too fast decline in water potentials (~3.3 MPa) were watered with 150 mL to achieve similar water potentials at peak drought in both treatments. From then on, water was also completely withheld in the long drought treatment. The total drought duration was 18 days and 51 days, including on average 4.2 and 6.6 days below the P_12_ threshold, in the short drought treatment and the long drought treatment, respectively. On DOY 233, all trees were re-watered with 1.5 L of water, matching the previously tested soil water-holding capacity in each pot. Re-watering was performed gradually over 3 hours, closely monitoring SWC, until a constant SWC was reached, which indicated fully saturated soils. From then on, all trees were regularly watered (3 × 150 mL/day). During the time from the start of the long drought treatment (DOY 184) to the end of the 12-day recovery period (DOY 244), the tree water deficit was assessed, and needle water potential was monitored frequently. After the end of the intense monitoring of the 12-day recovery period, the trees were removed from the chambers and placed in a separate compartment of the greenhouse. The trees were watered regularly, and air temperature was between 13°C and 20°C. On DOY 319, 75 days after re-watering, the trees were transported to the synchrotron facility for micro-CT imaging.

### 2.3 Tree- and leaf-level water fluxes

Prior to the initiation of the long drought treatment, the leaf-level gas exchange of all trees was measured to ensure comparability with a LI-COR 6400 (see **Supplementary Figure S1**). After the long drought treatment was initiated, the trees were moved into separate tree chambers. This previously tested system allowed us to derive tree- and leaf-level gas exchange dynamics (Birami et al., 2020; Rehschuh et al., 2020; Gattmann et al., 2021). The system comprised 20 tree chambers, with one being used as an empty chamber to offset potential fluctuations in gas exchange fluxes (on average 0.03 ± 0.07 mmol mol^−1^ H_2_O). As one of the trees died during the experiment, it was removed from the analysis, resulting in 18 tree chambers (n = 4 control, n = 5 short drought, and n = 9 long drought). Each chamber was constructed using a transparent aboveground compartment that encloses the tree shoot, sealed from an opaque belowground compartment that encloses the lower part of the stem and root system. A water-cooling system ensured that air temperatures within the shoot compartment were maintained between 22°C and 25°C during daytime. Note that the temperature of the coolant was maintained between 17°C and 19°C in order to avoid condensation within the shoot compartment. Air temperatures were derived in each shoot compartment by fast-response thermocouples (5SC-TTTI-36-2 M, Newport Electronics GmbH, Deckenpfronn, Germany). PAR was measured within each chamber using pre-calibrated photodiodes (G1118, Hamamatsu Photonics, Hamamatsu, Japan). Soil moisture was continuously logged (TS 107, Campbell Scientific, Inc., USA; EC 5, Meter Group, USA) per pot.

We constantly supplied aboveground chamber compartments with air from a compressor adding predefined CO_2_ (418 ± 3 µmol mol^−1^) and H_2_O concentrations (8.3 ± 0.6 mmol mol^−1^). Each of the aboveground compartments received an air flow of c. 14 L min^−1^. We determined the exact flow rate per chamber at the end of the experiment in order to calculate gas exchange fluxes. We assessed the absolute concentrations of CO_2_ and H_2_O in the supplying air (W_supply_) using a gas analyzer (Li-840, LI-COR, Lincoln, NE, USA). We then derived the differences between the supply air and the sample air (W_sample_) using a differential gas analyzer (Li-7000, LI-COR, Lincoln, NE, USA). Once every 40 min, we measured each aboveground compartment, and the duration of each single measurement cycle was 120 sec, and we used the last 10 sec to calculate gas exchange fluxes. We derived VPD and transpiration fluxes at the canopy level in order to assess the overall tree-level recovery potential. We favored canopy fluxes, as we could not resolve needle area on a daily basis, we assessed drought-induced needle shedding only at the end of the experiment. Additionally, we provided leaf-level transpiration rates normalized by the needle area remaining on the tree to also examine the proportion in recovery attributable to needle area loss. We derived tree- and leaf-level fluxes, as well as chamber-specific VPD, as follows.

VPD in kPa was calculated from saturation vapor pressure (e_s_) and actual vapor pressure (e_a_):


(1) $$e_{s}=0.6108\;\text{exp}\left(\frac{17.27\;T}{T+237.3}\right)$$

with air temperature (T) in °C, and


(2) $$e_{a}=\frac{AH\;P}{0.62198+AH}$$

with absolute humidity (AH) in g m^−3^ and atmospheric pressure (P) in kPa,


(3) $$VPD=e_{s}-\;e_{a}$$

Transpiration at canopy level (*E*_c_) in mol H_2_O tree^−1^ s^−1^ was calculated as follows:


(4) $$E_{c}\;=\;\dot{m}\frac{\left({\text{W}}_{\text{sample}}–{\text{ W}}_{\text{supply}}\right)}{\left(1\;–{\text{ W}}_{\text{sample}}\right)}$$

and transpiration at leaf area (E_la_) in mol H_2_O m^−2^ s^−1^,


(5) $$E_{la}=\;\dot{m}\frac{\left({\text{W}}_{\text{sample}}–{\text{ W}}_{\text{supply}}\right)}{area_{needle}\left(1\;–{\text{ W}}_{\text{sample}}\right)}$$

where $\dot{\text{m}}$ is air mass flow (mol s^−1^), W_supply_ is the absolute [H_2_O] in air supply, W_sample_ is the absolute [H_2_O] in sample air, and area_needle_ is the total remaining needle area per tree in m^2^.

Canopy conductance (*g*_c_) in mmol tree^−1^ s^−1^ was calculated accordingly:


(6) $$g_{c}\;=E_{c}\frac{\left(1000\;–\;\left(\frac{W_{needle}\;+\;W_{sample}\;}{2}\right)\right)}{\left(W_{needle}\;–\;W_{sample}\right)}$$

and stomatal conductance in mmol m^−2^ s^−1^,


(7) $$g_{s}\;=E_{la}\frac{\left(1000\;–\;\left(\frac{W_{needle}\;+\;W_{sample}\;}{2}\right)\right)}{\left(W_{needle}\;–\;W_{sample}\right)}$$

where W_needle_ is the needle saturated H_2_O vapor pressure, derived from saturated vapor pressure (kPa) at the prevailing T_air_ (°C) and atmospheric pressure P (kPa). This approach of calculating *g*_c_ and *g*_s_ neglects boundary layer conductance, which should be negligible under high mixing conditions inside the chamber (Birami et al., 2020).

The gas exchange data were processed by first excluding periods of obvious system malfunctioning. Then, the data for daytime conditions (6 am to 7 pm) were filtered. Further, outliers (1.5 times outside the interquartile range per chamber) were removed, which resulted in a data set containing 92% of the overall daytime data.

### 2.4 Tree water deficit

To observe stem diameter changes induced by variations in water supply, trees were equipped with a high-resolution dendrometer (DD-S, Ecomatik, Dachau, Germany). Before transferring the trees to the chambers, 10 trees were monitored: five in the long drought treatment and five in the control treatment. Once in the chambers, all trees were equipped with dendrometers. The dendrometer time series were processed and standardized using the “treenetproc” R package (version 0.1.4; Knüsel et al., 2021). The processed dendrometer time series were divided into growth- and water-related components of stem radius variation based on the zero-growth concept (Zweifel et al., 2016). According to this concept, growth begins when the previous maximum stem diameter is surpassed and ends when the stem starts to decrease in size. Any diameter changes below the previous maximum stem diameter were identified as a period of TWD. TWD was hence calculated as follows:


(8) $$\text{TWD }=\;\left(d_{max}\;–\;d\right)$$

and TWD_rel_ (%)


(9) $$TWD_{rel}\;=100\left(\frac{\left(d_{max}\;–\;d\right)}{d_{max}}\right)\;$$

where *d* is the current absolute stem diameter and *d*_max_ is the maximum diameter measured for an individual tree in the past, which sets the theoretical maximum diameter under conditions of fully hydrated tissues. To integrate the drought stress over time and derive a stress dose signal, the cumulative tree water deficit (TWD_P12_; mm) was derived as the integral of the TWD, i.e., the sum of TWD from days where water potential was below the P_12_ threshold (12% loss of hydraulic conductivity), which was derived based on leaf water potential measurements and hydraulic vulnerability curves (see next sections).

### 2.5 Water potential

Midday water potential (Ψ_md_) was measured frequently every second to third day throughout the experiment using C-52 psychrometers connected to a PSYPRO water potential system (Wescor, Logan, UT, USA). The advantage of this approach was the small sample size needed, causing little disturbance to our gas exchange measurements. Per tree, only two to three needles were needed, which we cut into small pieces and placed into the sample holders. The psychrometer chambers were then sealed, and measurements were taken after the temperature and water vapor between the needle sample and chamber air had equilibrated. The required equilibration time (15 minutes to several hours) was estimated based on previous tests and expected water potentials. For the calibration of C-52 psychrometers, filter paper disks (⌀ 5 mm) were soaked in osmolality standard solutions (Optimole™, ELITechGroup, Puteaux, France) with a defined water potential of −0.25 MPa, −0.7 MPa, and −2.5 MPa and carefully loaded into the sample psychrometer. As calibration and measurement temperature may differ throughout the experiment, a manual temperature-dependent conversion was used to calculate water potentials from the µV readings of the PSYPRO. PSYPRO water potential measurements were used to derive minimum leaf water potential (Ψ_min_), calculate the drought stress duration (dP_12_) by counting the days each tree spent under a vulnerability threshold (P_12_; see “Hydraulic vulnerability” section), and calculate the cumulative water potential (Ψ_cum_) by summing the PSYPRO water potential values during the respective drought period after subtracting the baseline water potential values of the control treatment.

### 2.6 Hydraulic vulnerability

The hydraulic vulnerability to embolism formation was assessed prior to the experiment on stems of six randomly selected individuals using the Cavitron technique (Cochard, 2002) to infer the P_12_, i.e., the pressure at which 12% of hydraulic conductivity is lost. From that information, together with the water potential, the stress duration, i.e., the days each treatment has spent under this threshold (dP_12_), was calculated. To avoid clogging of tracheids by resin, the bark was completely removed. Further, sampling artifacts were excluded by multiple re-cutting of the distal ends under water to a sample length of c. 28 cm. A custom-built rotor of 28 cm in diameter, inside a centrifuge (Sorvall RC-5; Thermo Fisher Scientific, Waltham, MA, USA), was used, and the sample ends were placed in transparent reservoirs filled with a solution of distilled, filtered (0.22 µm), and degassed water enriched with 0.0005 v/v% “Micropur Forte MF1000F” (Katadyn, Products, Wallisellen, Switzerland). The measurements followed the standard method outlined by Beikircher et al. (2010). In brief, the hydraulic conductance (*k*) of the sample was measured at successively reduced xylem pressures (*P*; MPa), which was induced through a step-wise increase in the rotational speed. Percentage loss of conductivity (PLC) was then calculated as follows:


(10) $$PLC\;=\;100\left(1-\frac{k_{t}}{k_{i}}\right)$$

where *k_i_* is the initial hydraulic conductance and *k_t_* is the hydraulic conductance at the respective *P*. Vulnerability analyses were performed by plotting PLC *versus* P. Fitting of the vulnerability curve including the water potential at 12% and 50% loss of conductivity (P_12_ and P_50_, respectively), the lower and upper confidence intervals, as well as the slope of the curve, was performed with the software package “fitplc” in R using the Weibull model (Duursma and Choat, 2017). One model was fitted, and the sample replicates were included as a random factor (**Supplementary Figure S3**).

### 2.7 Micro-CT imaging

Synchrotron micro-CT scans enable the visualization of embolized tracheids in secondary xylem. Scans were conducted at the TopoTomo beamline of the IPS imaging cluster at the KIT Light Source in Eggenstein-Leopoldshafen on 15–19 November 2021, approximately 75 days post-drought. To assess differences in embolism spread among different plant parts and between treatments, the apical shoot (mean diameter 10 ± 2 mm), stem middle (mean diameter 22 ± 3 mm), and the first-order order root at the transition to the stem (mean diameter 17 ± 5 mm) were scanned. The potted trees, previously measured in the chamber system, were transported to the beamline just before the measurements took place. Before the micro-CT measurements were conducted, the xylem water potential of end-twigs was determined using a Scholander pressure chamber. All trees were previously well-watered, and the mean water potential was 0.7 ± 0.2 MPa. As the trees were too large for the measurement stage, samples were prepared immediately before the micro-CT scans, as follows. The samples were excised at least 5 cm above and below the point of measurement to avoid the artificial formation of embolism during sample preparation (Tyree and Zimmermann, 2002; Beikircher et al., 2010). Cut ends were then wrapped into cling film and immediately fixed into a custom-built holder on the measurement stage of the beamline. The white-beam spectrum at the beamline, delivered by a 1.5-T bending magnet, was filtered using 1-mm Al. The X-ray projections of the tomography scan were recorded using an indirectly converting X-ray area detector composed of a 200-µm-thick LuAG: Ce scintillator, an Optique Peter white beam microscope providing a magnification of ×2, and a PCO.DIMAX camera with 2,016 × 2,016 pixels. The effective pixel size was 6.11 µm, and the effective field of view was 12.31 mm. For apical shoots and main roots, 180° scans with 3,000 projections were chosen, and for stems, 360° scans with 6,000 projections at an enlarged field of view (by placing the rotation axis to the outermost detector column). The distance between the sample and the detector (propagation distance) was set to 85 mm. Scan time per sample was approximately 3 min with a frame rate of 40 to 70 frames s^−1^. Then, the scanned region was marked and remeasured once the sample was fully dehydrated (bench drying on a hot plate) to obtain a reference scan in which all conduits were embolized. After the manual selection of suitable slices of the tomographic reconstruction, the ImageJ/Fiji image-processing software (National Institutes of Health, Bethesda, MD, USA) was used to calculate the percent loss of water-conductive area (PLA). Brightness and contrast were adjusted individually for each sample to improve visualization of water- *versus* air-filled conduits. Via color thresholding, the embolized conduit areas of both the initial and reference scans were calculated. The percentage of air-filled *versus* water-filled stem xylem conduits was assessed by excluding the pith, the primary xylem, and the resin channels. In a few samples, which were partly blurred or cut due to geometrical restrictions, only a representative part of the scan, present in both sample and reference scans, was used. Exemplary micro-CT images can be seen in the **Supplementary Data**. Please note that in scans of apical shoots and roots, colors were inverted during initial processing to enhance visibility. As a result, embolisms appeared in lighter gray, while water-filled conduits appeared in darker gray. In contrast, the scans of stems display the opposite pattern (for example images, see **Supplementary Figure S2**).

### 2.8 Needle biomass and needle area

From the total needle biomass, sampled per tree on 22 November, 2 days after micro-CT measurements, the total needle area per tree was derived. Additionally, all shed needles per tree were collected. All needle biomass samples were oven-dried for 48 h at 60°C, and dry weight was determined. The total needle area of each tree was then derived from needle biomass and predetermined specific needle area (g DW cm^−2^), as follows. On a subsample of fresh needles, specific needle area was measured using an area meter (Li-3,100, LI-COR, Lincoln, NE, USA), and dry mass was determined. The total needle area per tree was then determined by multiplying the needle dry biomass by the specific needle area. The same calculation was used to assess loss in needle area due to needle shedding.

### 2.9 Data analysis and statistics

All data processing and statistics were conducted using Rstudio version 2023.06.0 + 421 (R Core Team, 2023).

Soil water content, the TWD_rel_, and water potential are presented as daily means and standard error (SE) per treatment derived from the daily averages per tree. Treatment differences over time were compared at specific time points during the experiment (the last 3 days before the start of the long drought, the last 3 days before the start of short drought treatment, the last 3 days of drought, and the last 3 days of recovery) using linear mixed effect models with the “lme4” R package (adding individuals as a random factor) and *post-hoc* Tukey’s tests. Minimum water potential (Ψ_min_) per treatment was compared with a Student’s t-test by comparing the minimum water potential of all trees within a drought treatment to the other drought treatment.

The recovery index for each of the two stress treatments was derived as follows:


(11) $$R_{x}\;=\left(\frac{x_{recovery}}{x_{control}}\right)\times 100$$

where R_x_ is the recovery index of *E* or *g*_c_ given in %, x_recovery_ is the mean value of the parameter measured (here *E*_c_ or *g*_c_) per tree during the last 8 days of the recovery period, and x_mean control_ is the mean value of the parameter from control trees over the same period. First, R_x_ was calculated for each tree in the short drought and long drought treatments, and then the treatment average (± SE) was derived. Next, linear regression models with treatment as a fixed effect were used to assess associations with variation across different treatments. However, this effect was only marginally significant, and the primary explanatory variable was the drought stress metric in all cases. Therefore, linear regression models were fitted across treatments to evaluate the relationship between recovery indices and drought stress metrics (Ψ_min_, dP_12_​, TWD_P12_​, Ψ_cum_​, and PLA). To assess model fit, multiple linear regression models were compared using Akaike’s information criterion (AIC) and R^2^. Treatment differences of these indices, minimum water potential and days under P_12_, cumulative water potential, TWD_P12_, and PLA per treatment were compared by ANOVA and *post-hoc* Tukey’s tests.

## 3 Results

### 3.1 Drought development

**Figure 2:**
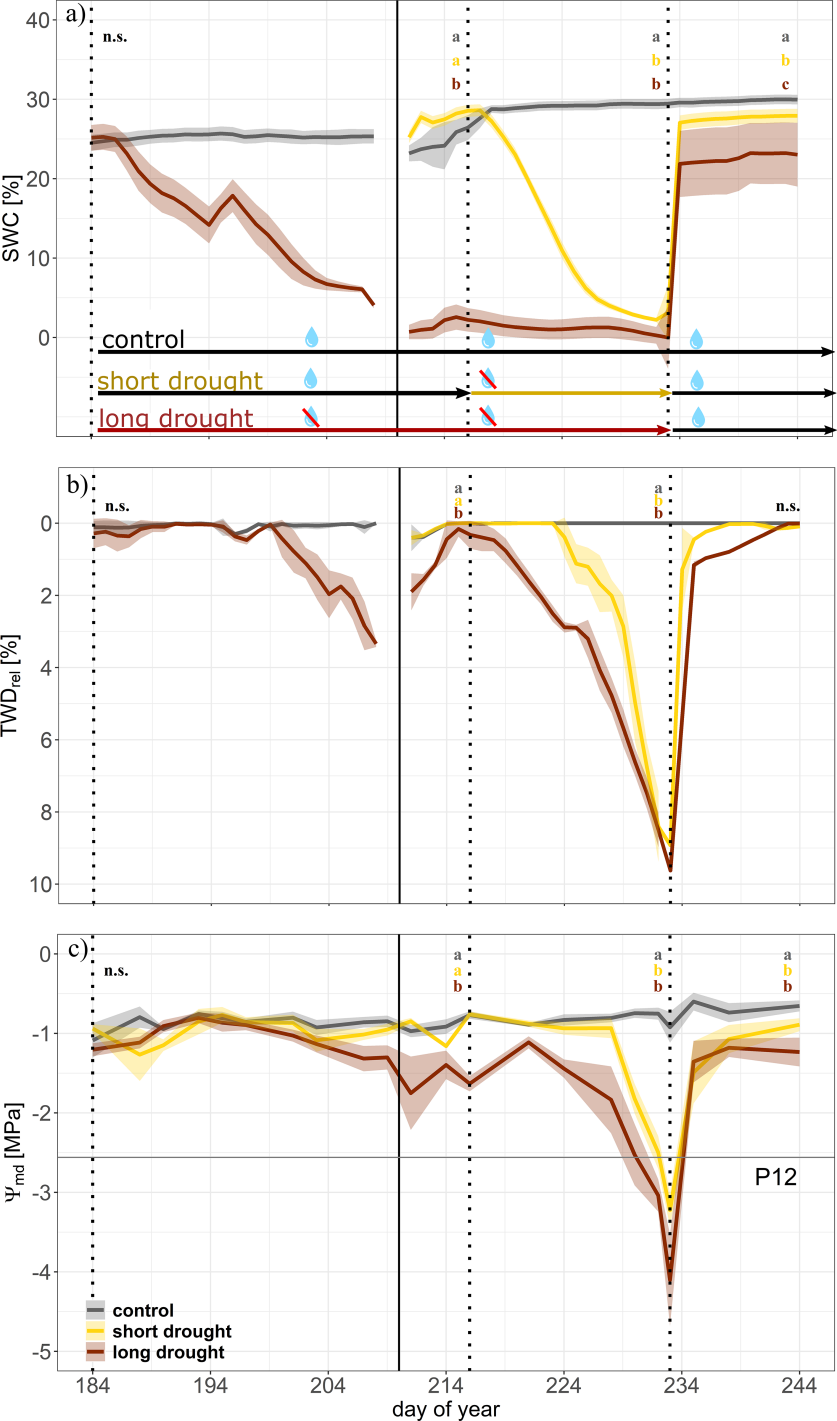
Time series of drought development during the drought and recovery experiment in Norway spruce. Dynamics of soil water content (SWC; **(A)**, tree water deficit (relative to maximum stem diameter; TWD_rel_; **(B)**, and midday water potential (Ψ_md_; **(C)** for control (gray, n = 4), short drought (yellow; n = 5), and long drought (dark red; n = 9) trees. Data are averages per day and treatment. The shaded areas represent the standard error per treatment. The first vertical line indicates the start of the long drought stress treatment, the second solid line indicates the transfer of trees into the individual tree chambers, the third vertical line indicates the start of the short drought stress treatment, and the fourth vertical line indicates the start of the recovery period. Well-watered conditions are indicated by black arrows between vertical lines; the yellow arrow indicates the drought period for the short drought treatment and the red arrow for the long drought treatment. Trees of the long drought treatment were supplied with 150 mL of water on days 194, 209, 210, and 214 to prevent a too fast decline in leaf water potential. During the transition of trees into the individual chambers, the number of trees equipped with dendrometers changed in each treatment (before, n = 5 for each control and long drought; after, n = 4 for control, n = 5 for short drought, and n = 9 for long drought). This transition led to a break in continuous data of SWC and TWD. The water potential threshold at 12% loss of hydraulic conductance (P_12_) is represented by the horizontal line in panel **(C)**. Letters indicate significant differences between treatments at the end of each period (n = 3 days) following linear mixed effect models and *post-hoc* Tukey’s test (p < 0.01).

Soil water content (**Figure 2A**) in both treatments declined once irrigation was reduced. Daily-averaged SWC in the short drought treatment decreased at a constant rate from 28.4% ± 1.3% to a minimum of 4.3% ± 0.8% before re-watering, whereas SWC in the long drought treatment declined at 26% ± 0.9% and remained consistently low (2.5% ± 0.9%) for 24 days before re-watering. Following re-watering, a sharp increase in daily-averaged SWC to well over 23% was observed. Leveling off while further water was supplied indicated full saturation of soils on the second day of recovery and onward (**Figure 2A**). During the drought period, VPD in the tree chambers remained consistently higher in the long drought treatment (c. 4–6 kPa) compared to that in the control but reached 6 kPa in the short drought treatment only at the end of the drought period (**Supplementary Figure S3**). Differences in VPD arose due to lower transpiration rates of the trees, as supplied humidified air could not fully compensate for the impact of reduced transpiration rates, resulting in drier air in the chambers, particularly during drought stress when stomata were fully closed.

### 3.2 Dynamics of hydraulic parameters during drought and re-watering

We found a strong signal of the tree water deficit in both drought treatments (**Figure 2B**) leading to a maximum stem shrinkage of 8.5% ± 0.3% at the end of the short drought and 9.6% ± 0.2% at the peak of the long drought. Stem shrinkage was highly similar between samples within treatment, as tree diameters were similar in trees previously selected for the experiment. The trees under short drought exhibited a tree water deficit for 15 days, while the long-drought trees were under tree water deficit for 48 days, more than three times as long, resulting in a larger cumulative TWD. During short drought, drought-induced cumulative stem shrinkage reached 4.83 ± 0.5 mm, while a long drought resulted in a cumulative shrinkage of 14.65 ± 0.7 mm. When considering only the cumulative tree water deficit on days when water potential reached the P_12_ threshold (TWD_P12_), short drought caused a cumulative shrinkage of 3.8 ± 1.3 mm, compared to 9.3 ± 1.4 mm during long drought. Note that the short, intermitted recovery phase of TWD in the long drought trees, during placement of the trees in the gas exchange chambers, was due to extra water supplied, which allowed for a small rehydration but not a full recovery; for instance, we did not find a recovery in Ψ_md_.

A similar water stress progression became evident for the dynamics of Ψ_md_ (**Figure 2C**). During the drought treatment, Ψ_md_ of the long drought trees was lower than that of control trees on 36 days, whereas Ψ_md_ of trees under short drought was below that of the control on 19 days. Trees in the long drought treatment experienced a Ψ_md_ below the P_12_ (−2.56 MPa) for 6.60 ± 1.0 days on average, longer than in the short drought treatment where a Ψ_md_ below P_12_ was reached for approximately 4.20 ± 0.7 days on average. This was also reflected in a tendency for a lower Ψ_min_ before re-watering with −4.10 ± 0.2 MPa in the long drought trees compared to −3.22 ± 0.58 MPa in the short drought trees (t-test, p = 0.3). These minimum Ψ_md_ values were close to the confidence intervals of the average P_50_ value derived from the vulnerability curves (P_50_: −3.85 [−3.67, −4.06] MPa). Accumulated water stress during short and long droughts, when Ψ_md_ was lower than control values, resulted in a Ψ_cum_ of −7.99 ± 0.86 and −14.91 ± 1.18 MPa, respectively.

Following re-watering, we found a sharp increase in TWD reaching control values in both treatments, although it took longer for trees after the long drought stress to reach control values than after the short drought. We found a similar result for Ψ_md_, but leveling off at −1.15 ± 0.09 MPa for short drought and −1.26 ± 0.19 MPa for long drought; both remained significantly below the control at the end of the recovery period [Tukey’s honestly significant difference (HSD), p > 0.01].

### 3.3 Persistence of drought-induced embolism in specific organs

**Figure 3:**
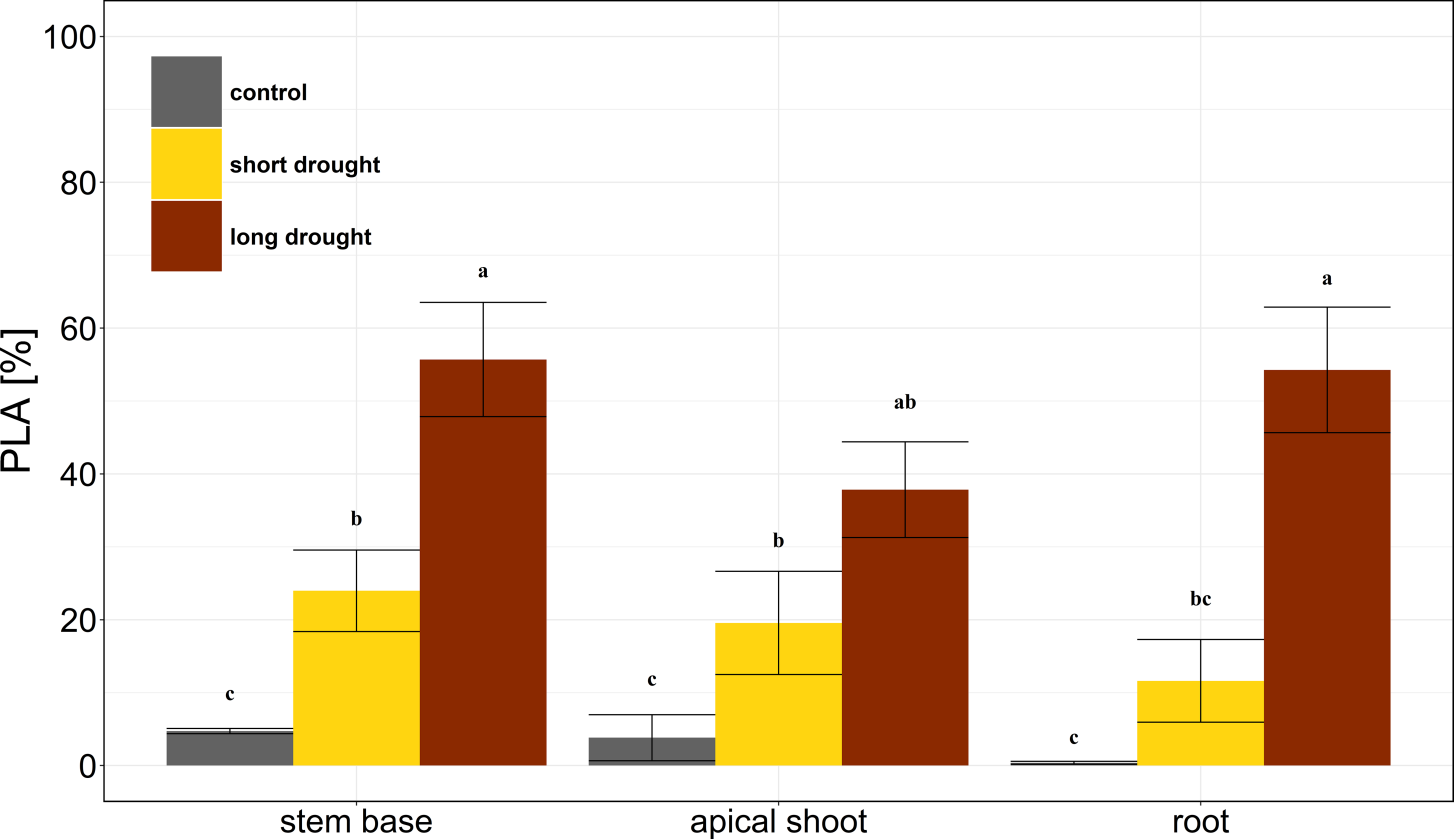
Embolism in Norway spruce after drought and subsequent recovery. The percentage loss of conductive area (PLA) is shown for stem base, apical shoot, and first-order root in the control (gray, n = 4), short drought (yellow, n = 5), and long drought (dark red, n = 9) treatments. Mean and SE. Black letters indicate significant differences (p < 0.05) in PLA across both treatment and plant parts as measured by ANOVA followed by a combined *post-hoc* Tukey’s test.

The stress dose was reflected in the persistence of xylem embolism 75 days after re-watering. We found that the largest PLA in the long drought treatment with 48.5% ± 9.5% averaged over all measured plant parts (**Figure 3**). In contrast, the short drought treatment showed a much lower PLA with 18.4% ± 6.1% (**Figure 3**). In comparison, the native PLA of control trees was on average 2.9% ± 1.2%. We found no differences in PLA levels between plant parts in the respective treatments. The individual tree-specific PLA 75 days after re-watering correlated equally well with the lowest water potential measured (Ψ_min_; R^2^ = 0.68), as well as the duration below the P_12_ (dP_12_; R^2^ = 0.68), cumulative water potential (Ψ_cum_; R^2^ = 0.62), and the cumulative tree water deficit from days when water potential was below the P_12_ (TWD_P12_; R^2^ = 0.69; **Supplementary Figure S5**).

### 3.4 Transpiration and canopy conductance

**Figure 4:**
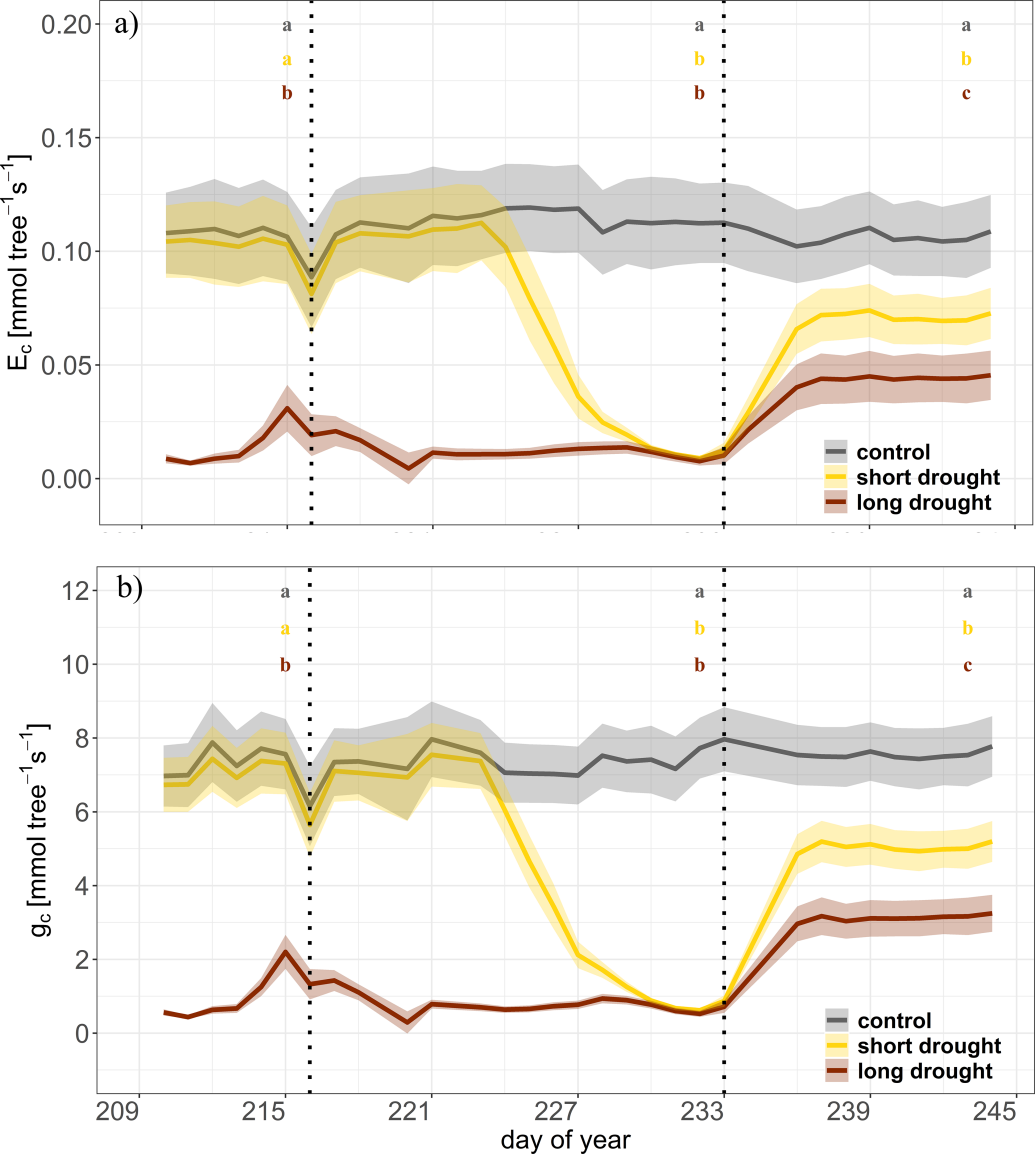
Time series of canopy transpiration (*E*_c_) and canopy conductance (*g*_c_) during the drought and recovery in Norway spruce. Dynamics of canopy transpiration (*E*_c_, **A**), and canopy conductance (*g*_c_, **B**) for control (n = 4, gray), short drought (n = 5, yellow), and long drought (n = 9, dark red) trees in the individual tree chambers. Data are averages per day and treatment. The shaded areas represent the standard error per treatment. The first vertical line indicates the start of the short drought stress treatment, and the second vertical line indicates the start of the recovery period. Letters indicate significant differences between treatments at the end of each period (n = 3 days) following linear mixed effect models and *post-hoc* Tukey’s test (p < 0.01).

**Figure 5:**
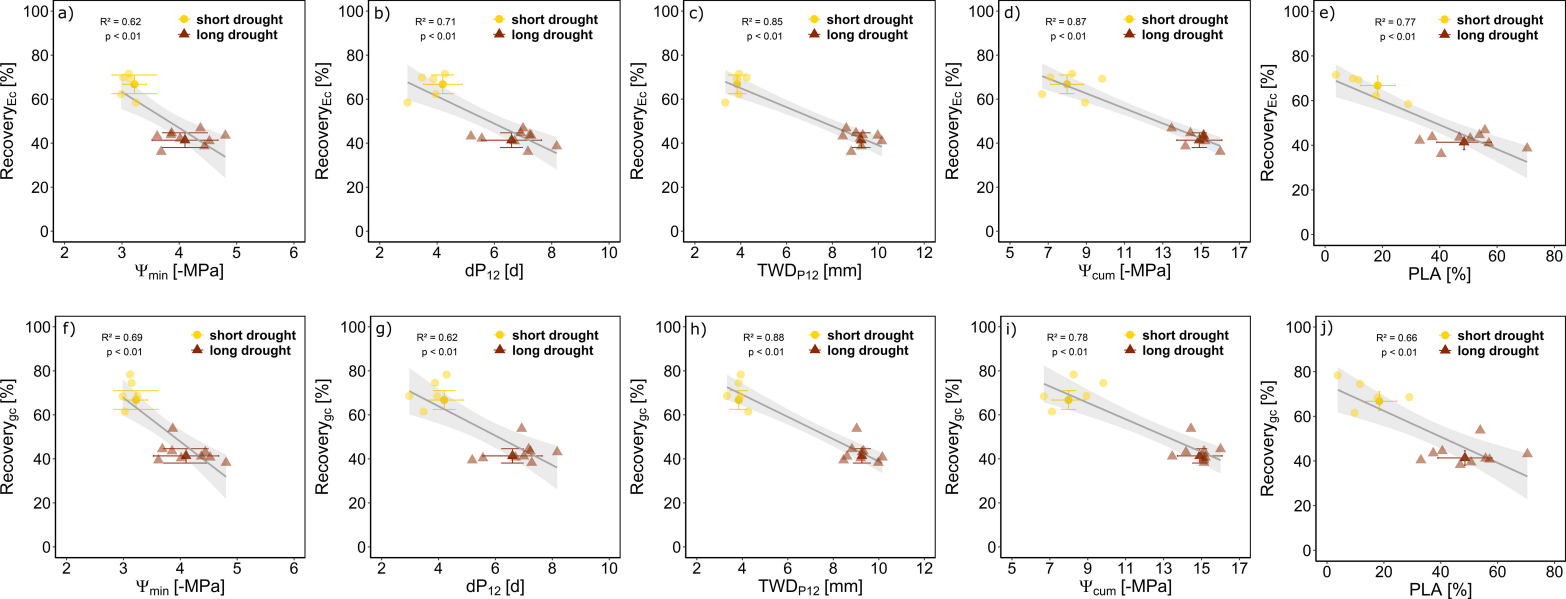
Recovery of *E*_c_ and *g*_c_ in relation to different drought stress measures for Norway spruce. Relative recovery per tree following the release from short (n = 5, yellow) or long drought (n = 9, dark red) is given in percentage compared to the control minimum water potential measured (Ψ_min_; **A, F**) on days below P_12_ **(B, G)**, tree water deficit accumulated during days where water potential was below P_12_ (TWD_P12_; **C, H**), cumulative water potential (Ψ_cum_; **D, I**), and the average percent loss of water conductive area over all measured plant parts (PLA; **E, J**). Data are presented as mean ± SE, and individual data points per tree (lower opacity) are the mean of the last 5 days of recovery. Linear regression lines (gray) ± SE (light gray) are given with R^2^ and p-values.

Transpiration and *g*_c_ were monitored starting on DOY 210 (i.e., before the start of short drought stress) for 34 days, including the 12-day recovery period. Due to the preceding drought in the long drought treatment, the rates of *E*_c_ and *g*_c_ were already close to zero (except for a slight increase on DOYs 214 and 215 due to additional water supply; see “Experimental Design” section). In the short drought treatment, *E*_c_ and *g*_c_ strongly declined to near zero on DOY 224 and onward (**Figures 4A, B**). Following re-watering, clear differences were found between the short and long drought treatments with lower *E*_c_ and *g*_c_ in the trees recovering from long drought (**Figure 4**; Tukey’s HSD, p < 0.01). The short-drought trees showed a higher recovery rate, reaching approximately 69% of *E*_c_ compared to control trees, while in the long drought, the recovery was much lower and remained at approximately 45% (**Figures 4A**, **5**). After the short drought, *g*_c_ recovered to 66% of the control rates but only to 41% following the long drought (**Figures 4B**, **5**). Even though SWC during recovery was found to be lower in the short and long drought treatments than in the control treatment (**Figure 2A**), this obviously had no effect on tree water exchange rates, as pots were fully saturated and SWC at similar levels did not limit *E*_c_/*g*_c_ prior to stress.

We observed needle shedding in both drought treatments. Trees under short drought shed 12% ± 2% and trees under long drought shed 32% ± 3% of needle biomass. This translated into a loss of needle area of 8% in the short drought treatment and 30% in the long drought treatment (**Table 1**). To exclude the loss of needle area from gas exchange recovery, we derived leaf-level transpiration (*E*_la_) and stomatal conductance (*g*_s_). We found an overall higher recovery at the leaf level. For *E*_la_, the recovery was approximately 74% following a short drought and 60% following a long drought; for *g*_s_, it was approximately 72% after a short drought and 59% after a long drought (**Supplementary Figure S6**). This shows that after accounting for needle shedding, recovery was still incomplete, pointing toward intrinsic hydraulic constraints induced by stress.

**Table 1:** Needle biomass and needle area of Norway spruce trees.

Treatment	Biomass		Needle area		
	Needles	Shed needles	Needle loss	Needle area	Needle area loss
	(g)	(g)	(%)	(m^2^)	(%)
Control	25.8 ± 4.7	1.2 ± 0.5	4.8 ± 2.2	0.102 ± 0.019	0.6 ± 0.4
Short drought	18.3 ± 2.3	2.6 ± 1.1	12.4 ± 2.1	0.094 ± 0.010	7.8 ± 2.1
Long drought	14.4 ± 2.9	6.8 ± 4.3	32.1 ± 3.0	0.072 ± 0.014	29.4 ± 8.4

Needle biomass, shed needles, and the percentage needle loss for control trees and trees after short and long droughts. Total needle area and the percentage needle area loss for control trees and trees after short and long droughts. Values are given in mean ± SE.

### 3.5 Link of drought dose and hydraulic damage and recovery

We assessed the potential of different stress indicators, including stress intensity, duration, and dose, as well as post-drought PLA, in predicting the recovery rates of *E*_c_ and *g*_c_ post-drought. First, we assessed how well minimum Ψ, drought duration (dP_12_), and stress dose indicators (TWD_P12_ and Ψ_cum_) were related to the post-drought measured PLA. We found a clear relationship of all four indicators with PLA (**Supplementary Figure S5**), underlining their suitability as drought-recovery proxies. Further, we wanted to assess how well the drought duration, inferred from dP_12_, and stress dose, inferred from TWD_P12_ and Ψ_cum_, in comparison to Ψ_min_ or PLA, can describe the recovery delay of *E*_c_ and *g*_c_. We found all five drought-stress indicators to well describe recovery rates (**Figure 5**). The lowest explanatory power (lowest R^2^ and highest AIC; **Supplementary Table S1**) appeared when using minimum Ψ_min_ to predict recovery of *E*_c_, indicating that more factors than stress intensity play a role. For *g*_c_, recovery Ψ_min_, PLA, and Ψ_cum_ showed a similar explanatory power (**Figure 5**, **Supplementary Table S1**). The stress dose metrics appeared as best proxies for drought recovery of *E*_c_ (TWD_P12_ R^2^ = 0.85 and Ψ_cum_ R^2^ = 0.87) and *g*_c_ (TWD_P12_ R^2^ = 0.88 and Ψ_cum_ R^2^ = 0.78), also supported by the lowest AIC of the models (**Supplementary Table S1**). The advantage of using TWD_P12_ over dP_12_ or Ψ_cum_ as a drought-recovery indicator is that it provides a continuous signal, and no destructive sampling is needed. Additionally, the strong linear relationship observed between Ψ_md_ and TWD (R^2^ = 0.87, p < 0.001) shows that stem shrinkage is directly related to hydraulic stress (**Supplementary Figure S7**), underscoring the potential to use TWD to relate stress impacts and recovery rate.

## 4 Discussion

### 4.1 Drought stress dose as key factor in explaining hydraulic impairment

We addressed how differences in drought stress dose affected the post-drought recovery dynamics in 3-year-old *P. abies* trees. Trees in the long drought treatment were 2.5 days longer below the P_12_ threshold than trees in the short drought treatment, resulting in a larger accumulation of xylem embolism, even though additional watering on DOY 214 shortly recharged some water storage in the long drought treatment (**Figure 2B**) but did not lead to a significant recovery in SWC, Ψ, *g*_c_, or *E*_c_. This watering delayed a too rapid decline in Ψ, which was necessary to allow similar dehydration levels in the short and long drought treatments. Once no water was supplied and Ψ thresholds related to the onset of xylem embolism were exceeded, dehydration rates accelerated. In agreement with previous studies in mature Norway spruce, this led to a rapid loss of hydraulic conductivity (Arend et al., 2021). Comparing patterns in juvenile to mature trees may be biased as age-related differences in hydraulic vulnerabilities may exist due to differences in xylem structure (Domec and Gartner, 2003). In our study, we found the difference in Ψ_min_ at the end of the drought to be relatively small (short drought, −3.2 ± 0.2 MPa; long drought, −4.1 ± 0.6 MPa), but as these Ψ values are related to the steep part of the hydraulic vulnerability curve, the derived PLC appears remarkably different. Estimating PLC from these Ψ_min_ would result in a PLC of 22% in the short drought compared to a PLC of 58% in the long drought treatment. It is worth mentioning that these estimates of PLC based on Ψ_min_ are consistent with the range of hydraulic vulnerability curves reported for Norway spruce in previous studies (Tomasella et al., 2018; Rosner et al., 2019; Arend et al., 2021; Knüver et al., 2022). Comparing these Ψ_min_-based estimates of PLC to our actual measurements of the embolized xylem area conducted 10 weeks after the drought revealed similar patterns with a PLA of 18% in the short drought treatment and 49% in the long drought treatment. However, PLA and derived PLC cannot directly be compared, as a loss in hydraulic conductivity does not necessarily transfer into a loss of conduit area. For instance, in Scots pine, the reported PLA was much lower than the PLC at the end of the drought (Rehschuh et al., 2020). Conduits of different growth years and seasons can differ in their transport capacity and pit architecture, affecting overall conductivity. Also, partial refilling of embolized conduits, for instance, found after winter embolism in Norway spruce trees (Mayr et al., 2014) and white spruce (Laur and Hacke, 2014), or growth of new functional xylem between the start of the recovery period and micro-CT measurements, could have affected the PLA observed here. However, based on previous studies that found no evidence of xylem embolism refilling in conifers after the release from drought (Rehschuh et al., 2020; Gauthey et al., 2022) and as the relative treatment difference was similar between PLC and PLA, we assume that the observed PLA 10 weeks post-drought largely reflects the drought-induced losses in xylem functioning.

Thus, we suggest that the larger PLA post-drought in the long drought treatment corresponded to the longer drought duration (i.e., time under the P_12_ threshold). Trees of the long drought treatment maintained a more negative Ψ_md_ over a longer time, as drought progression was slower and intermitted by additional water supply. However, over time, stress accumulated, visible in a higher PLA as well as a larger cumulative Ψ and cumulative TWD_P12_. A larger TWD_P12_ is related to a longer duration under embolism-forming water stress in which the trees experienced a reduction in tree water reserves and hence stem shrinkage of the elastic tissues due to cuticular water loss. The utilization of these stem water reservoirs results in further dehydration (Zweifel et al., 2016), leading to irreversible damage of distal organs including needle shedding (Nadal-Sala et al., 2021), triggering the collapse of elastic cells and further inducing embolism spread to proximal organs (Mantova et al., 2022; Andriantelomanana et al., 2024).

### 4.2 Embolism levels in plant segments and needle shedding affected by drought stress

The hydraulic segmentation theory posits that distal organs are more susceptible to cavitation than central plant parts, thereby protecting the core of the hydraulic system from malfunction (Johnson et al., 2016). Supporting this theory, several studies have demonstrated differences in the hydraulic vulnerability of various plant organs (Rodriguez-Dominguez et al., 2018; Bourbia et al., 2020; Harrison Day and Brodribb, 2023), with distal segments such as apical shoots often showing greater vulnerability compared to stems. In contrast, other research suggests that differences in hydraulic vulnerability between plant segments, particularly stems and woody roots, are minor and highly variable across species (e.g., Losso et al., 2019). Our findings align with this latter perspective. Specifically, we observed no differences in embolism spread among the X-rayed plant segments, even under prolonged drought conditions. This result is consistent with that of a previous study on five tree species, which reported significant convergence in the drought vulnerability of stems and roots to cavitation (Peters et al., 2021a). A relatively uniform distribution of embolism across the measured plant segments as observed here indicates that the different tissues of Norway spruce trees were dehydrating at a similar rate in both treatments. Further, the differences in embolism spread among treatments were clearly related to the duration below a critical water potential (dP_12_) irrespective of the plant segment measured.

The observed embolism spread also agreed well with the observed needle shedding at the end of the experiment. Approximately one-third of the total needle area was shed in the long drought treatment, which was about twice as much as under short drought. Needle shedding has been observed to occur in conifers during drought, likely due to tissue dehydration, which prevents cuticular water loss and potentially contributes to drought acclimation (Nadal-Sala et al., 2021). However, in conifers, needle shedding can also indicate a loss in tree vigor, as replacing the lost needle tissue is carbon-demanding and thus highly costly to the tree (Song et al., 2022). Such stress-induced tissue damages as observed here reduce the overall carbon uptake and growth potential of the trees (Galiano et al., 2011; Trugman et al., 2018; Nadal-Sala et al., 2024).

### 4.3 Post-drought recovery progression

The pace of recovery depends on the physiological impacts of the drought. While gas exchange can recover quickly after mild drought, gas exchange may only partially recover after severe drought, as structural damages, such as impaired vascular function or collapsed cell walls, can occur (Brodribb and McAdam, 2013; Ruehr et al., 2019). It has been suggested that this is primarily due to the accumulation of drought-induced hydraulic damage (Ruehr et al., 2019; Rehschuh et al., 2020). After re-watering, we found an instant, almost complete recovery of Ψ_md_ and TWD. Indeed, a fast initial regaining of partial hydraulic functioning post-drought has been reported for a large range of species including *Eucalyptus viminalis* (Tonet et al., 2024), *Pinus halepensis* (Wagner et al., 2023), *Pinus sylvestris* (Rehschuh et al., 2020), and *P. abies* (Hájíčková et al., 2021). This was contrasted by a much slower, incomplete recovery of tree-level water loss and canopy conductance. After a first quick increment during the 3 days following re-watering, a plateau was reached with lower values in the long drought compared to the short-drought trees. While ABA has been shown to limit the initial 1–4 days of recovery (Ruehr et al., 2019), other factors are responsible for the incomplete recovery of leaf-level water fluxes. In our study, we found a persisting lower Ψ_md_ in the short and long drought treatment trees compared to the control. This lower Ψ_md_ could result from pronounced xylem embolism reducing tree water transport (Rehschuh et al., 2020) and consequently inducing partial closure of stomata due to the effects of reduced leaf hydration on stomatal guard cell turgor (McAdam and Brodribb, 2015) and limiting recovery of leaf-level transpiration to 60% of control values. At the canopy level, gas exchange was additionally limited by losses in needle area due to drought-induced leaf shedding. Although trees in the long drought treatment have lost 32% of their needles, canopy transpiration rates recovered to 41% of control values. This highlights the non-linear effect of leaf shedding on gas exchange, as conifers typically shed older needles (Nadal-Sala et al., 2021), which contribute less to overall gas exchange than current-year needles.

At both treatments, the tree- and leaf-level hydraulic impairment and damage are critical factors delaying post-stress recovery (Choat et al., 2018; Brodribb et al., 2020). Embolisms formed during periods of extreme water deficit can impair the ability of plants to transport water, thereby limiting the restoration of water potential, transpiration, and stomatal conductance. Furthermore, stomatal malfunctioning, such as delayed reopening or impaired stomatal control post-drought, has also contributed to the slow recovery of transpiration (McAdam and Brodribb, 2016) as observed here. Additionally, it is possible that the remaining needles suffered from partial tissue damage and functional impairment that reduced their photosynthetic capacity or hydraulic efficiency (Blackman et al., 2009), potentially due to oxidative stress, protein degradation, or other cellular damages caused by drought (Flexas et al., 2012). Root system impairment, such as reduced root hydraulic conductivity or damage to fine roots and root shedding often in concert with leaf shedding, may further limit water uptake (Anderegg et al., 2016). Thus, impaired root functionality (Brunner et al., 2015; Peters et al., 2021a), which is only slowly established again post-drought (Mackay et al., 2020), could further exacerbate the effects of needle loss. An overall reduced hydraulic functionality was reflected in non-fully recovered water potentials alongside a persistent impairment of stomatal conductance in Norway spruce, which limits carbon uptake and may delay the growth of new functional tissues and hence whole-tree recovery. Considering that the drought-induced embolism in Norway spruce were persisting in roots and stems until the end of the growing season, repair and/or new growth may be shifted to the next growing season. This highlights the potential of drought legacy impacts to prevail into the following year (Trugman et al., 2018).

### 4.4 Assessing drought stress dose as a predictor of recovery

We assessed the potential of different stress metrics to predict the recovery potential of Norway spruce trees post-drought. Loss of hydraulic conductivity (i.e., PLA or PLC) has previously been shown to be related to post-stress recovery potential (Ruehr et al., 2019; Rehschuh et al., 2020; Wagner et al., 2023). Loss of hydraulic conductance integrates the severity of the drought stress signal over time, with a larger PLA the longer the time spent under the P_12_ threshold, as found here (**Supplementary Figure S5**). However, measurements of hydraulic conductance are always destructive, often error-prone (Burlett et al., 2022), and/or expensive (e.g., micro-CT, Savi et al., 2017). Thus, other metrics of drought stress intensity are frequently used. Often the absolute minimum water potential is applied to characterize the severity of stress (Bhaskar and Ackerly, 2006; Martínez-Vilalta et al., 2021), which can then be linked to the recovery potential. However, this has limitations (Zang et al., 2014a, b), as it does not address the duration that the trees experience such negative tensions and hence does not necessarily relate to PLA or PLC. Hence, we explored the use of different metrics that integrate stress intensity over time.

Measures of drought dose related to recovery success reported in the literature are the cumulative Ψ (Zang et al., 2014a, b), which integrates Ψ over time, and cumulative growth responses after successive droughts (Navarro-Cerrillo et al., 2018; Gazol et al., 2020). Hence, in order to capture the full drought dose, frequent or continuous measurements are needed. Although Ψ_cum_ showed to be a reliable measure for stress dose in our study, using the cumulative Ψ as drought proxy is challenging, as measurements are typically elaborate and destructive and would require continuous Ψ measurements, but novel approaches have emerged, which may make this more feasible (Blanco and Kalcsits, 2021). In our study, we measured needle water potential using the PSYPRO on two to three needles every 2–3 days—a frequency that is rather challenging to achieve, even under controlled experimental conditions. Moreover, these Ψ measurements are typically non-continuous and represent conditions of one moment of the day. Thus, in order to make it feasible to assess the drought dose, not only in controlled experiments but also in the field, our results suggest using high-resolution point dendrometer measurements to inform about stress dose. Measurements of TWD have been found to be closely related to Ψ, providing a reliable approach to infer hydraulic stress in our study (**Supplementary Figure S7**), as well as in other studies (Cochard et al., 2001; Steppe et al., 2006; Drew et al., 2011; Dietrich et al., 2018; Peters et al., 2021b; Lauriks et al., 2022; Ziegler et al., 2024). Therefore, the integration of TWD measurements, particularly cumulative TWD, can complement traditional water potential assessments, offering a more accessible tool to evaluate the stress dose in real-time, especially in field conditions where continuous water potential measurements remain highly challenging. The strong linear relationship observed between water potential and TWD underscores the mechanistic link between these metrics (**Supplementary Figure S7**), demonstrating that TWD serves as a reliable proxy for changes in water potential by directly connecting stem shrinkage to hydraulic stress. This connection is further supported by the fact that the stress dose indices used here, the TWD_P12_ and Ψ_cum_, most effectively explained the delayed recovery of *E*_c_ and *g*_c_. Prolonged periods of dehydration as well as time spent below critical water potentials impair and eventually damage tree hydraulic processes, thereby hindering the tree’s ability to rapidly recover physiological processes post-drought. Thus, we suggest that integrating continuous measurements of TWD is highly suitable for detecting the stress dose and, consequently, the recovery potential in trees.

## 5 Conclusion

The drought dose, representing the integral of drought intensity over time, could well explain the retarded recovery post-drought and appears as a more promising approach than focusing on minimum water potential only. Specifically, the duration spent under critical thresholds, such as the onset of embolism, and the cumulative tree water deficit derived from point-dendrometer measurements were closely related to the degree of embolism formation, as well as the recovery potential of juvenile Norway spruce trees. However, it is important to note that the findings from this experiment are not directly transferable to adult trees, which possess a larger water storage capacity and may respond differently to drought stress. Moreover, a broader combination of stress intensity and duration would provide a more complete picture of the relevance of the stress dose as a recovery metric. This underscores the importance of time-explicit measurements of stress intensity and duration for determining the post-drought recovery potential of trees in the field. Given that water potential measurements are time-consuming, destructive, and challenging in mature forests, the cumulative tree water deficit derived from high-resolution point dendrometers could serve as a viable, non-destructive method for assessing the stress dose and recovery capacity of forests.

## Data Availability

The raw data supporting the conclusions of this article will be made available by the authors, without undue reservation.
