# Supplementary material for: Stress dose explains drought recovery in Norway spruce
Source: Front Plant Sci. 2025 Mar 6;16:1542301. doi: 10.3389/fpls.2025.1542301 (PMC11922940; doi:10.3389/fpls.2025.1542301)
Supplement: Supplementary file 1 [file DataSheet1.docx]

Stress dose explains drought recovery in Norway spruce - Supplement

**Timo Knüver*, Andreas Bär, Elias Hamann, Marcus Zuber, Stefan Mayr, Barbara Beikircher, and Nadine K. Ruehr**

*** Correspondence**Timo Knüver
[timo.knuever@kit.edu](mailto:timo.knuever@kit.edu)


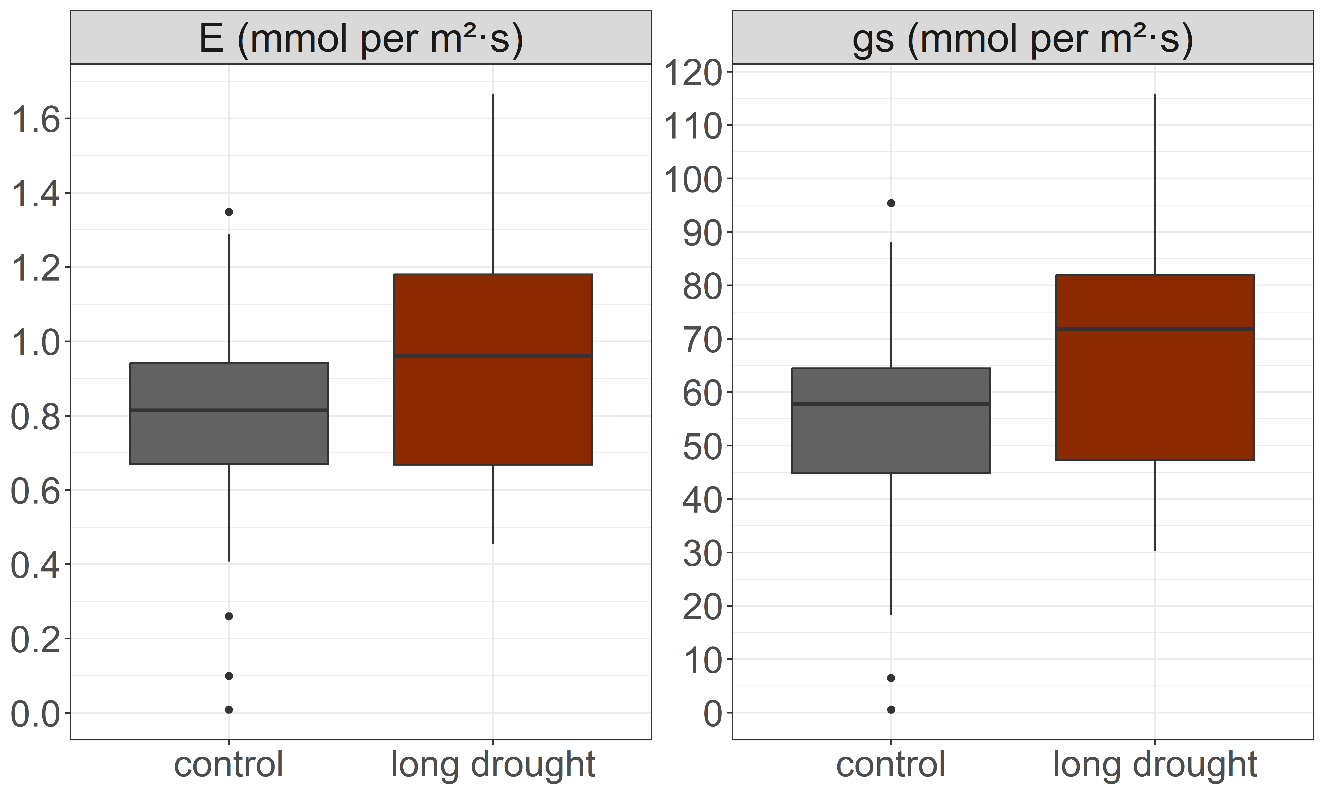


*Figure S1: Pre-stress baseline measurements of transpiration (E) and stomatal conductance (gs) of Norway spruce trees, measured with a LI-COR 6400 on doy 184, before drought in the long drought treatment was initiated (control: n = 35; long drought: n = 40.*

**
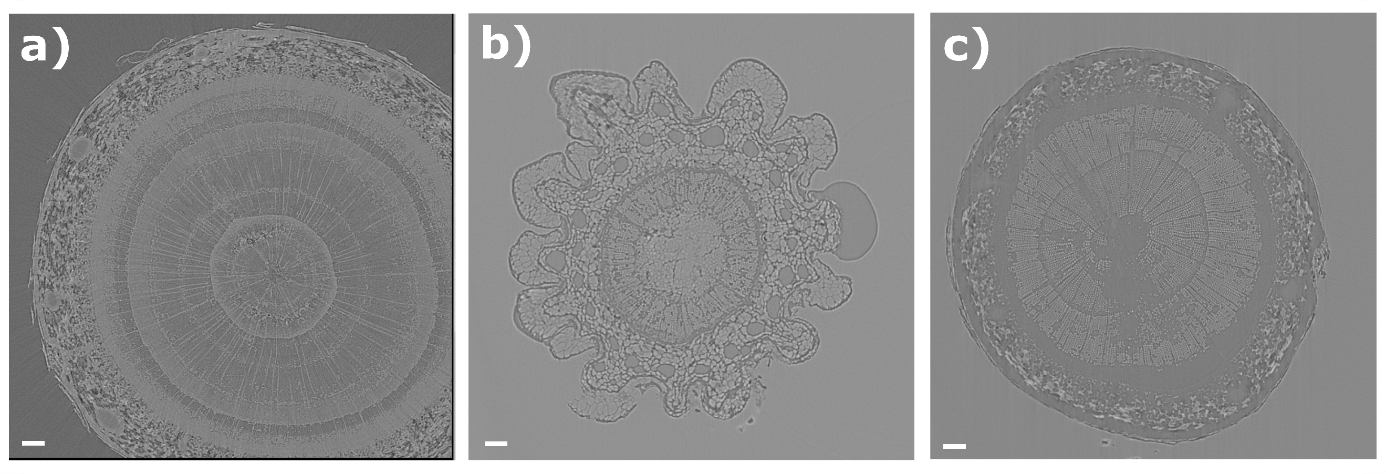
**

*Figure S2: Examples of visualization of xylem embolism in a stem middle (a), an apical shoot (b), and a 1^st^ order root (c). Reconstructed cross-sections of stems show embolized (dark grey) and water-filled (light grey) xylem conduits. In scans of apical shoots and roots, colors were inverted for visibility, with embolisms in lighter grey and water-filled conduits in darker grey, the reverse of scans for stems. White bars represent 1mm.*

**
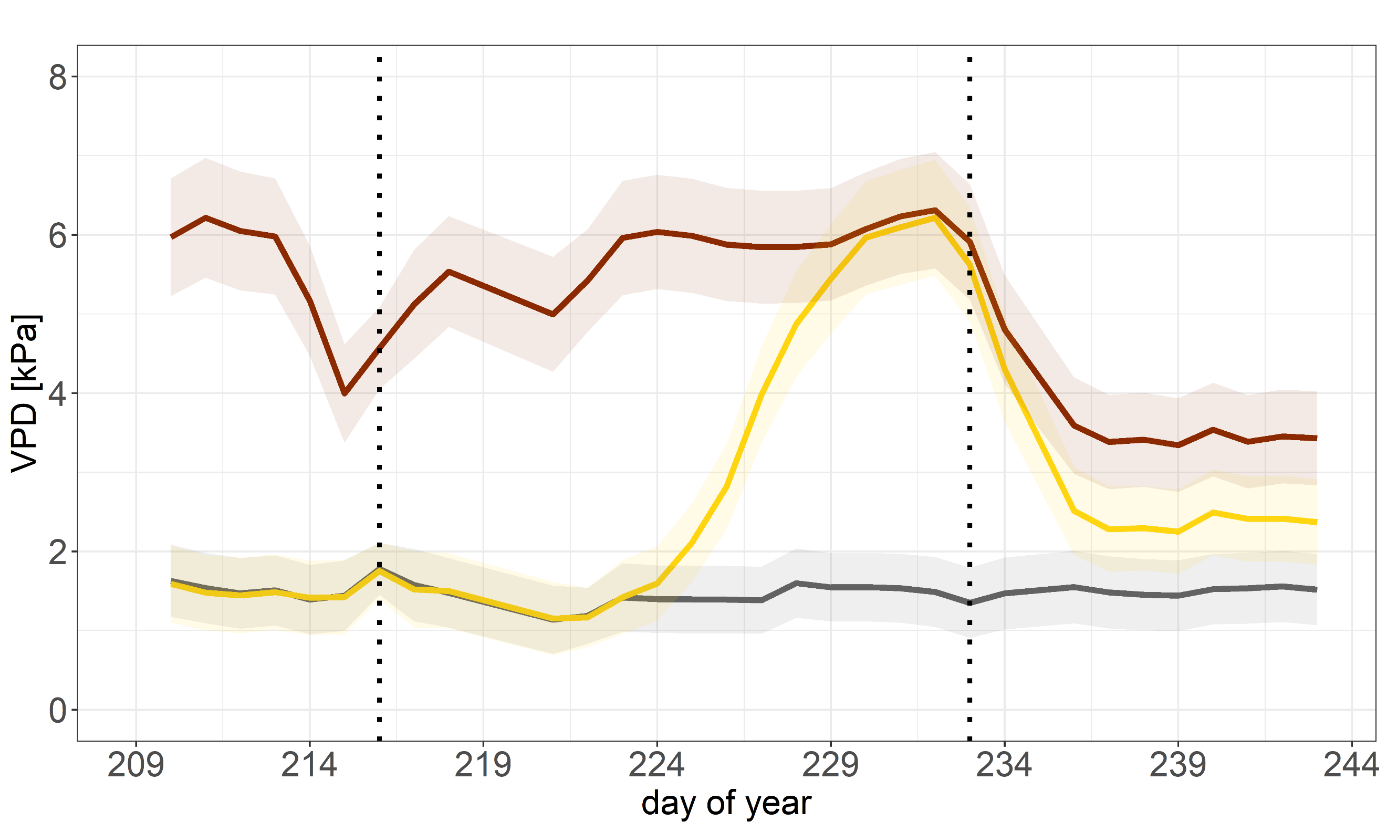
**

*Figure S3: Time series of water vapor pressure deficit in the individual tree chambers during the drought and recovery experiment in Norway spruce. Dynamics of VPD for control (n = 4, grey), short drought (n= 5, yellow) and long drought (n = 9, dark red) trees. Data are averages per day and treatment. The shaded areas represent the standard error per treatment. The first vertical line indicates the start of the moderate stress treatment, the second vertical line the start of the recovery period.*

***
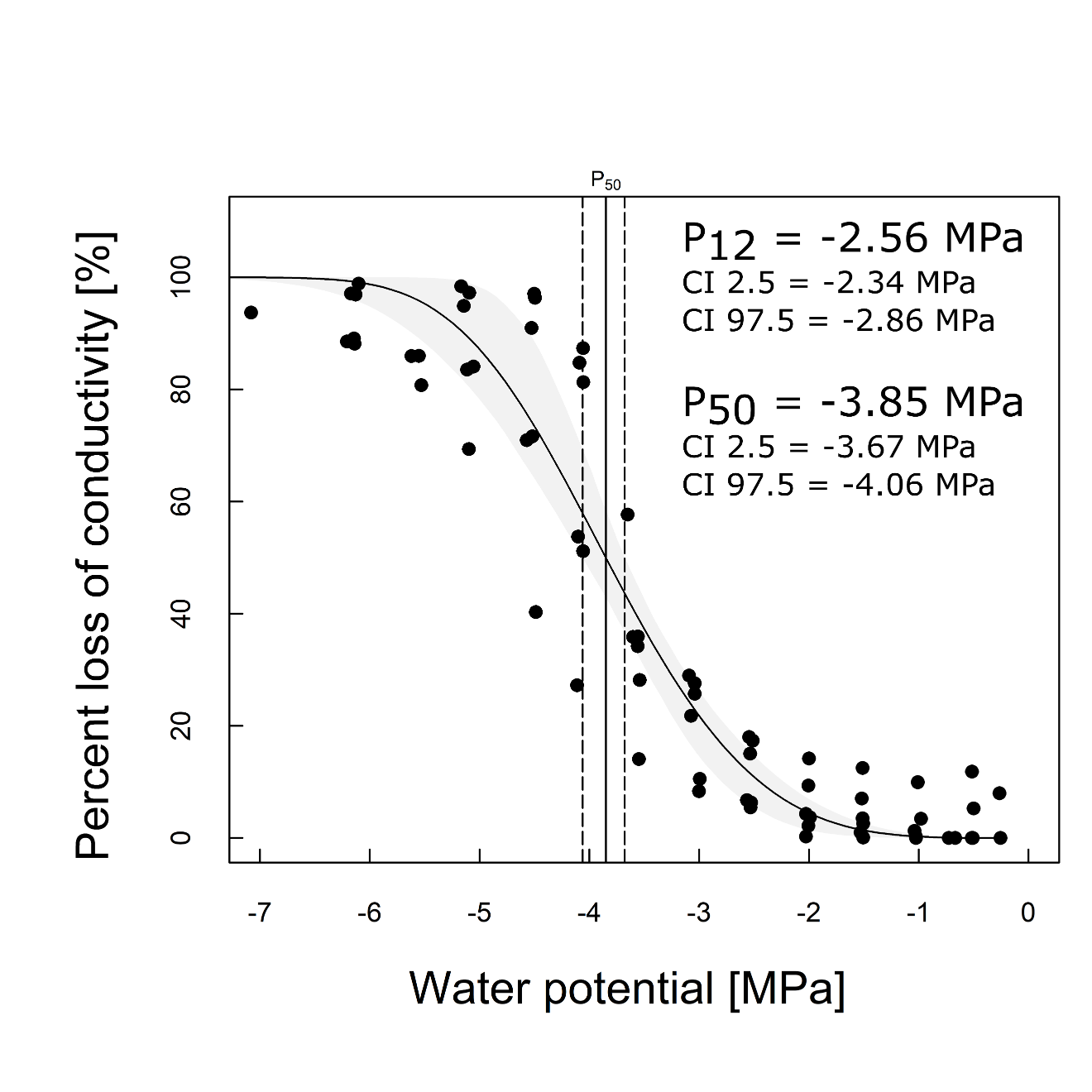
***

*Figure S4: Percentage loss of hydraulic conductivity versus xylem pressure of Norway spruce trees derived preliminary to the start of the experiment on 6 individuals. Vertical line indicates the water potential inducing 50% loss of conductivity (P_50_), vertical dotted lines show the 2.5% and the 97.5% confidence interval of the P_50_.Water potential inducing 12% loss of conductivity (P_12_) and P_50_ plus respective upper (97.5%) and lower (2.5%) confidence interval are shown in the upper right corner. Shaded area represents the 95% boot-strapped confidence interval for the fitted curve.*

**
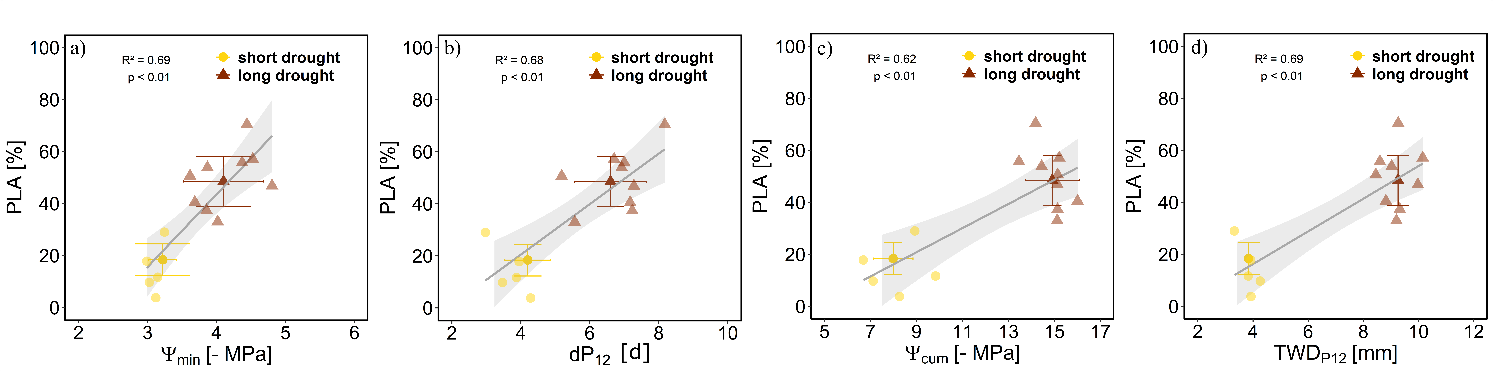
**

*Figure S5: Relationship of the percent loss in hydraulic conductive area (PLA) with the lowest water potential measured (Ψ_min_, a), days below P_12_ (b),cumulative water potential (Ψ_cum,_ c), and cumulative tree water deficit at days with a water potential under P_12_ (TWD_P12_, d) in Norway spruce trees with short (n = 5, points, yellow) and long drought (n = 9, triangles, dark red). Displayed point data is averaged ± SE. Linear regression lines (grey) ± SE (light grey) are shown, as well as r-squared and p-values.*

**
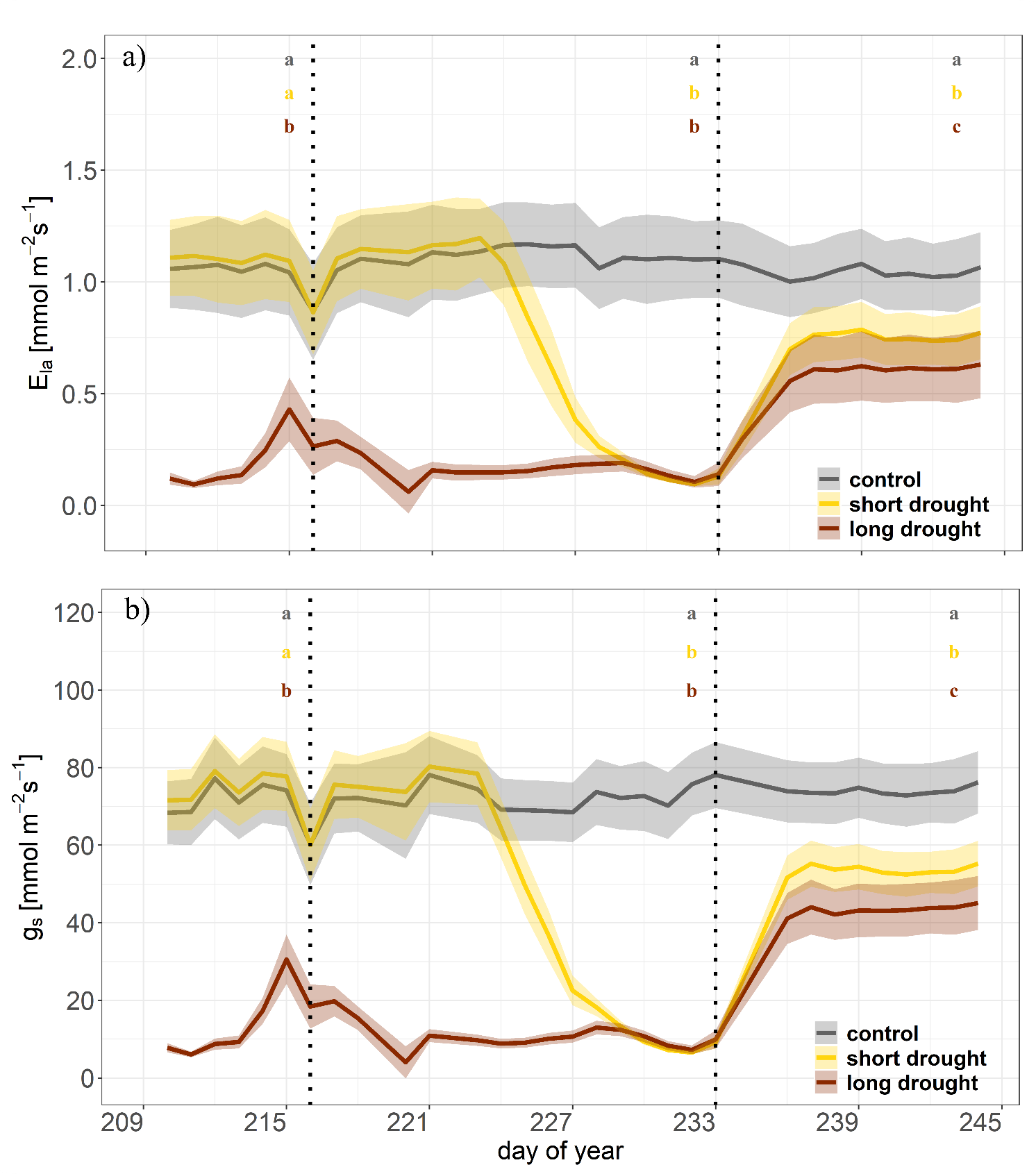
**

*Figure S6: Time series of leaf-level transpiration (E_la_) and stomatal conductance (g_s_) during the drought and recovery in Norway spruce. Dynamics of leaf-level transpiration (E_la_, a), and stomatal conductance (g_s_, b) for control (n = 4, grey), short drought (n = 5, yellow) and long drought (n = 9, dark red) trees in the individual chambers. Data are averages per day and treatment. The shaded areas represent the standard error per treatment. The first vertical line indicates the start of the moderate stress treatment, the second vertical line the start of the recovery period. Letters indicate significant differences between treatments at the end of each period (n = 3 days) following linear mixed effect models and post-hoc Tukey test (p < 0.01).*

*
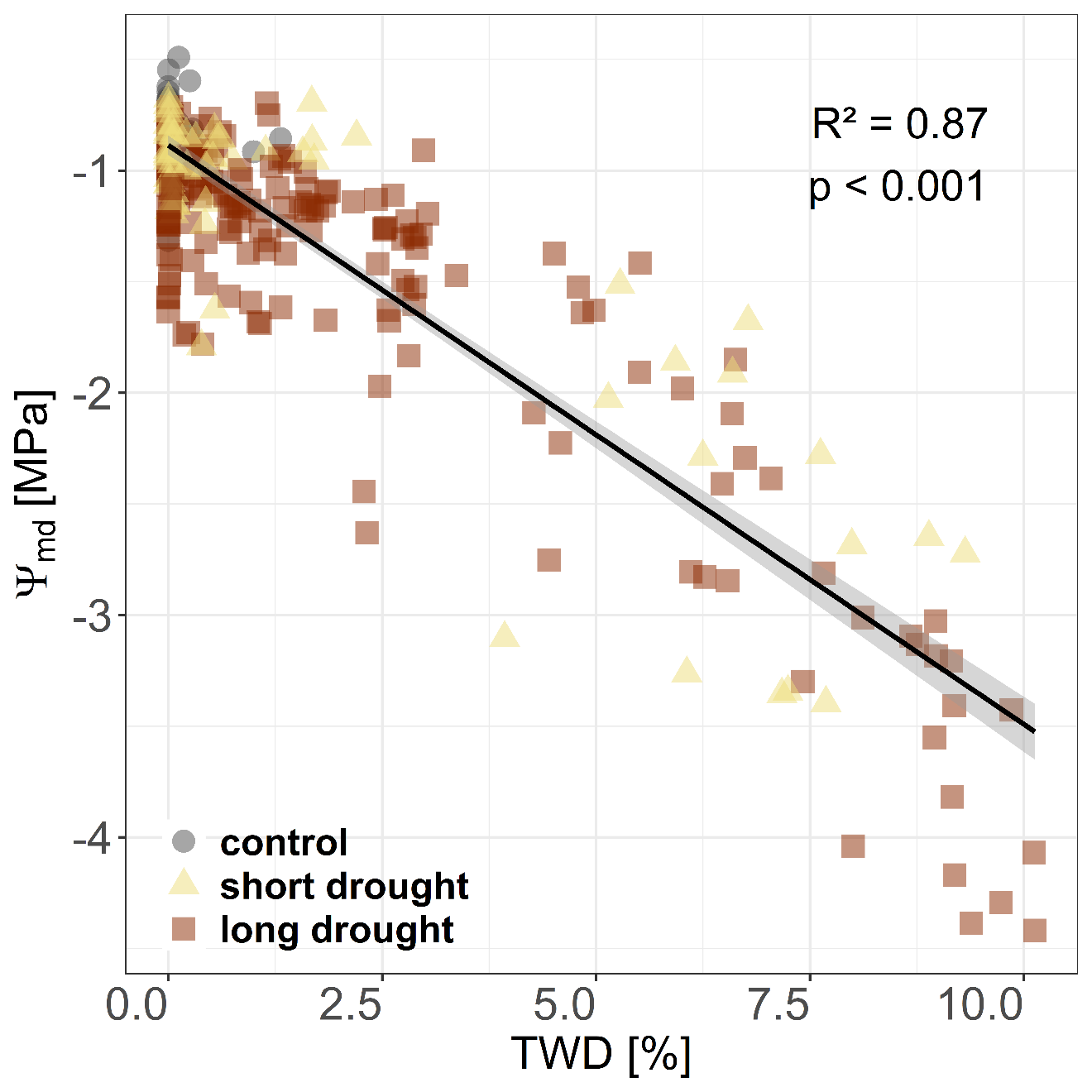
*

*Figure S7: Relationship of relative tree water deficit (TWD) and water potential (Ψ_md_) during a drought experiment on juvenile Norway spruce trees with a control (grey circles), a short drought (yellow triangles), and a long drought (red squares) treatment.*

*Table S1: Evaluation of best fit model to explain recovery percentages of Canopy transpiration and conductance. Linear regression models were applied and Akaike Information Criterion (AIC) and R-squared goodness of fit (R²) were used to assess the fit of five linear regression models for canopy transpiration (E_c_) and five linear regression models for canopy conductance (g_c_), allowing for a comparison of model performance within each group.*

| **Model** | **R²** | **AIC** |
| --- | --- | --- |
| **E_c_ ~ Ψ_cum_** | **0.87** | **87.44** |
| ***E*_c_ ~ TWD_P12_** | **0.85** | **88.77** |
| **E_c_ ~ PLA** | **0.77** | **95.10** |
| **E_c_ ~ dP12** | **0.71** | **98.36** |
| **E_c_ ~ Ψ_min_** | **0.61** | **102.22** |
| **Model** | **R²** | **AIC** |
| ***g*_c_ ~ TWD_P12_** | **0.88** | **90.57** |
| ***g*_c_ ~ Ψ_cum_** | **0.78** | **98.66** |
| ***g*_c_ ~ Ψ_min_** | **0.68** | **103.41** |
| ***g*_c_ ~ PLA** | **0.66** | **104.61** |
| ***g*_c_ ~ dP12** | **0.61** | **106.24** |
